# Healthcare access dimensions and uterine cancer survival: a national cancer database study

**DOI:** 10.3389/fonc.2023.1263371

**Published:** 2023-10-05

**Authors:** Mary Katherine Anastasio, Anjali Gupta, Tomi F. Akinyemiju, Rebecca A. Previs

**Affiliations:** ^1^Department of Obstetrics and Gynecology, Duke University Medical Center, Durham, NC, United States; ^2^Department of Population Health Sciences, Duke University School of Medicine, Durham, NC, United States; ^3^Stanford University School of Medicine, Stanford, CA, United States; ^4^Division of Gynecologic Oncology, Duke Cancer Institute, Durham, NC, United States; ^5^Labcorp Oncology, Durham, NC, United States

**Keywords:** uterine cancer, healthcare access, racial disparities, chemotherapy, radiotherapy

## Abstract

**Objective:**

Disparities exist throughout diagnosis, treatment, and survival for Black patients with uterine cancer. There is limited data on how several healthcare access (HCA) dimensions contribute to these disparities in patients with advanced stage uterine cancer.

**Methods:**

Using the National Cancer Database (NCDB), we identified patients aged 40-89 years with Stage III-IV uterine cancer between 2004-2015 who received chemotherapy and/or radiotherapy. Race/ethnicity were classified as non-Hispanic (NH)-Black, Hispanic, and NH-White. Variables defined in the NCDB were used to assess HCA affordability, availability, and accessibility. Kaplan-Meier estimates, log-rank test, and multivariable Cox proportional hazards models were used to analyze overall survival.

**Results:**

Of 43,134 patients, 78.8% of the cohort identified as NH-White, 15.3% NH-Black, and 5.9% Hispanic. NH-Black patients were the most likely to have type II (75.6% vs. 53.9% and 55.4%) and stage IV (40.8% vs. 30.7% and 32.3%) disease compared to NH-White and Hispanic patients. NH-Black patients were more likely than NH-White and Hispanic patients to have government funded insurance (58.6% vs. 50.3% and 50.4%), live in low-income areas (46.4% vs. 14.2% and 29.9%), and receive only chemotherapy (53.5% vs. 43.1% and 46.2%). Having private insurance and receiving treatment at an academic facility were positive predictors of survival. NH-Black patients had worse survival than NH-White patients after adjusting for clinical characteristics and healthcare access dimensions (HR 1.29; 95% CI 1.24, 1.34).

**Conclusion:**

While HCA affordability and availability predicted survival in patients with advanced stage uterine cancer, additional factors contribute to racial disparities. Compared to NH-White patients, NH-Black patients had more aggressive disease, received only chemotherapy rather than combined therapy, and had worse survival regardless of cancer subtype. Additional dimensions of healthcare access must be explored to remedy uterine cancer disparities.

## Introduction

Uterine cancer is the fourth most common and sixth most lethal cancer among women in the United States (1). While most present with disease at an early-stage, generally curative with surgery alone, 10-15% present with advanced disease and poor prognosis (2). Patients with early-stage disease have a roughly 95% overall survival (OS) rate, while patients with locally advanced (stage III/IVA) and distant (stage IVB) disease have OS rates of 69.8% and 18.4%, respectively (3). Treatment options for advanced disease include chemotherapy, radiotherapy, or combined chemoradiation (4). Uterine cancer has historically been divided into two pathogenic types (5). Type I tumors are highly differentiated, low-grade tumors with a favorable prognosis and include well-differentiated endometrioid adenocarcinomas. Type II tumors are poorly differentiated, high-grade tumors with increased risk of recurrence and include serous, clear cell, and poorly differentiated endometrioid tumors.

The incidence of uterine cancer has been increasing, with the most significant increases observed in non-Hispanic Black, Hispanic and Asian women (6). The incidence rate of aggressive uterine cancer subtypes increased, particularly among non-Hispanic Black women (6). Uterine cancer mortality has also been increasing; the death rate for Black women is almost double the death rate of White women (7, 8). Black women have worse 5-year survival rates than White women for nearly every stage and histologic subtype of uterine cancer (9–13). Racial disparities persist across many aspects of uterine cancer care. Black women are less likely to receive appropriate evaluation of postmenopausal bleeding (14). Black and Hispanic women are more likely to present with advanced disease at diagnosis which contributes to a racial disparity in survival (10, 15), more likely to have type II histologies (9) and less likely to receive definitive surgery (10, 12), minimally invasive surgery (16), lymph node sampling or dissection (10), and guideline-concordant care (17).

When assessing disparities in uterine cancer care, the effects of differential access to money, resources, and health care on uterine cancer treatment and survival must be considered. Five dimensions of healthcare access (HCA) have been described by the Penchansky and Thomas framework (18), and operationalized by our team (19): affordability (capability to pay for services), availability (quantity, nature, and value of services), accessibility (service site), accommodation (service structure), and acceptability (patient experience, quality of patient–provider interaction). Among those with uterine cancer, lack of access to healthcare is associated with disparities in survival (9–13). Thus, identifying HCA factors which drive racial inequities in survival, particularly among those with advanced-stage disease and inherently lower survival rates, is imperative. The association of specific factors of HCA dimensions, such as facility type, SES, and insurance status, and survival has been demonstrated previously. Using variables measured in the NCDB, this study simultaneously evaluates the association of three HCA dimensions (affordability, availability, accessibility) estimable in the NCDB dataset with racial/ethnic disparities in survival among diverse patients with advanced stage uterine cancer.

## Methods

### Data source

We obtained data from the 2016 NCDB Participant User File for uterine cancer. The NCDB is a collaborative project of the American College of Surgeons and American Cancer Society. It encompasses over 70% of new cancer cases in the United States and Puerto Rico and contains sociodemographic data, including race/ethnicity, and clinical data including cancer stage, histology, and treatments (20). This study received Duke University Institutional Review Board approval (IRB#: Pro00102834).

### Study cohort

Our study cohort comprised of patients ages 40-89 years with stage III-IV uterine cancer diagnosed during 2004-2015 who received chemotherapy and/or radiotherapy. We excluded patients who had missing pertinent variables or sociodemographic characteristics. Race/ethnicity was defined as Non-Hispanic-White (NH-White), NH-Black, and Hispanic. Those of other or unknown races and ethnicities were excluded from the analysis.

### Study measures and covariates

Race/ethnicity was the primary exposure, and all-cause mortality was the primary outcome. Survival estimates were calculated using length of time from diagnosis to death or last encounter. Demographic and clinical covariates included age at diagnosis, Charlson-Deyo comorbidity score, cancer stage, receipt of chemotherapy and/or radiotherapy, surgery receipt, and cancer subtype (type I, type II, and other). Type I cancers included grade 1-2 endometrioid (9). Type II cancers included grade 3 endometrioid, serous, clear cell, mixed cell, and carcinosarcoma. Other histologic subtypes were categorized as ‘other’.

HCA affordability variables included insurance status (no insurance, private, and government), area-level education, and area-level income. Area-level education level was defined as the proportion of adults ≥25 years in the patient’s zip code that did not graduate high school and is presented as quartiles: ≥17.6%, 10.9% -17.5%, 6.3% -10.8%, and <6.3%. Median household income was estimated and categorized as quartiles: <$40,227, $40,227-$50,353, $50,354-$63,332, and ≥$63,333. Area-level education level and median household quartiles were predefined by the NCDB (21). Insurance status was provided in the NCDB using categories including not insured, private insurance/managed care, Medicaid, Medicare, and other government; the latter three were combined to create an overall “government” category for the purpose of this analysis. HCA availability was represented by cancer facility type (academic and non-academic). Variables for HCA accessibility included residence type (rural, urban, and metro) and “great circle” distance (crowfly) between patient residence and hospital location.

### Analytical plan

We described demographic, clinical, and HCA characteristics of our study cohort by race/ethnicity. We used Kaplan-Meier estimates, log-rank test, and multivariable Cox proportional hazards models adjusted for patient age, comorbidity score, year of diagnosis, cancer stage, cancer subtype, surgery receipt, chemotherapy receipt, and radiation receipt. We repeated these models in analyses stratified by cancer subtype. We ran a series of multivariable Cox proportional hazards models additionally adjusting for measures representing each HCA dimension: a) affordability; b) availability; c) accessibility; and d) all three. We used SAS 9.4 (SAS Institute, Cary, NC, United States) for analysis, and statistical significance was designated with two-sided p<0.05.

## Results

There were 43,134 patients with advanced stage uterine cancer included in this analysis: 78.8% NH-White, 15.3% NH-Black, and 5.9% Hispanic (**Table 1**). Approximately 71.6% of NH-Black patients were 60 years or older, compared to 65.5% of NH-White and 54.5% of Hispanic patients. Most patients were diagnosed with stage III disease (67.7%) and type II cancers (57.3%). NH-Black patients were the most likely to have type II cancer (75.6% vs. 53.9% and 55.4%) and stage IV disease (40.8% vs. 30.7% and 32.3%) compared to NH-White and Hispanic patients. Most patients underwent surgery (87.3%); NH-Black patients were less likely to undergo surgery than NH-White or Hispanic patients (81.9% vs 88.4% and 87.5%). Approximately 44.8% of patients received chemotherapy only, 14.9% received radiotherapy only, and 40.2% received chemotherapy and radiation. NH-Black patients were more likely to receive chemotherapy only (53.5% vs 43.1% and 46.2%) and less likely to receive combined chemoradiation (32.4% vs 41.7% and 40.8%) compared to NH-White and Hispanic patients.

**Table 1:** Sociodemographic and cancer characteristics stratified by race/ethnicity for Stage III-IV uterine cancer patients who received chemotherapy and/or radiotherapy.

Variable	Overall N = 43134 (%)	NH-White N = 34006 (78.8%)	NH-Black N = 6591 (15.3%)	Hispanic N = 2537 (5.9%)
Age (years)				
40-49	3426 (7.9)	2659 (7.8)	394 (6.0)	373 (14.7)
50-59	11332 (26.3)	9074 (26.7)	1476 (22.4)	782 (30.8)
60-89	28376 (65.8)	22273 (65.5)	4721 (71.6)	1382 (54.5)
Distance from Hospital (miles) (mean, SD)	28.3 (83.4)	30.5 (86.6)	20.6 (69.4)	20.2 (71.0)
Facility Type				
Academic	18942 (43.9)	13965 (41.1)	3624 (55.0)	1353 (53.3)
Not Academic	24192 (56.1)	20041 (58.9)	2967 (45.0)	1184 (46.7)
Charlson-Deyo Score				
0	32316 (74.9)	25959 (76.3)	4527 (68.7)	1830 (72.1)
1	8583 (19.9)	6375 (18.8)	1622 (24.6)	586 (23.1)
2	1697 (3.9)	1290 (3.8)	321 (4.9)	86 (3.4)
3+	538 (1.3)	382 (1.1)	121 (1.8)	35 (1.4)
Insurance				
Government	22226 (51.5)	17088 (50.3)	3861 (58.6)	1279 (50.4)
Private	19072 (44.2)	15768 (46.4)	2335 (35.4)	969 (38.2)
No insurance	1836 (4.3)	1150 (3.4)	397 (6.0)	289 (11.4)
Residence				
Rural	761 (1.8)	683 (2.0)	73 (1.1)	5 (0.2)
Urban	6206 (14.4)	5516 (16.2)	585 (8.9)	105 (4.1)
Metro	36167 (83.9)	27807 (81.8)	5933 (90.0)	2427 (95.7)
Income				
<$40,227	8648 (20.1)	4832 (14.2)	3057 (46.4)	759 (29.9)
$40,227-$50,353	9589 (22.2)	7697 (22.6)	1289 (19.6)	603 (23.8)
$50,354-$63,332	10041 (23.3)	8503 (25.0)	999 (15.2)	539 (21.3)
$63,333+	14856 (34.4)	12974 (38.2)	1246 (18.9)	636 (25.1)
No High School Degree				
>17.6%	9219 (21.4)	5047 (14.8)	2741 (41.6)	1431 (56.4)
10.9-17.5%	11296 (26.2)	8659 (25.5)	2150 (32.6)	487 (19.2)
6.3-10.8%	12411 (28.8)	10878 (32.0)	1157 (17.6)	376 (14.8)
<6.3%	10208 (23.7)	9422 (27.7)	543 (8.2)	243 (9.6)
Stage				
III	29197 (67.7)	23578 (69.3)	3902 (59.2)	1717 (67.7)
IV	13937 (32.3)	10428 (30.7)	2689 (40.8)	820 (32.3)
Receipt of Therapy				
Chemotherapy Only	19342 (44.8)	14648 (43.1)	3523 (53.5)	1171 (46.2)
Radiotherapy Only	6441 (14.9)	5178 (15.2)	931 (14.1)	332 (13.1)
Both	17351 (40.2)	14180 (41.7)	2137 (32.4)	1034 (40.8)
Surgery				
Yes	37658 (87.3)	30044 (88.4)	5395 (81.9)	2219 (87.5)
No	5476 (12.7)	3962 (11.7)	1196 (18.2)	318 (12.5)
Subtype^a^				
Type I	12187 (28.3)	10665 (31.4)	775 (11.8)	747 (29.4)
Type II	24726 (57.3)	18336 (53.9)	4985 (75.6)	1405 (55.4)
Other	6221 (14.4)	5005 (14.7)	831 (12.6)	385 (15.2)

a Type I histologies include grade 1-2 endometrioid (codes 8050, 8140, 8143, 8210-8211, 8260-8263, 8340, 8380-8384, 8560, 8570). Type II histologies include grade 3 endometrioid, serous (8441, 8460-8461), clear cell (8255, 8323), mixed cell (8310), and malignant Mullerian mixed tumors (MMMT) or carcinosarcoma (8950-8951, 8980-8981).

NH-Black patients were the least likely to have private insurance (35.4% vs. 46.4% and 38.2%) compared to NH-White and Hispanic patients. There were racial differences in measures of affordability; almost half (46.4%) of all NH-Black patients were in the lowest income quartile, compared to only 14.2% of NH-White and 29.9% of Hispanic patients. While 27.7% of NH-White patients lived in areas among those with the highest education, only 8.2% of NH-Black and 9.6% of Hispanic patients lived in these areas. There were also racial differences in measures of accessibility; when stratified by race/ethnicity, NH-White patients lived farther from their hospital than NH-Black and Hispanic patients (mean 30.5 vs. 20.6 and 20.2 miles) and were less likely to live in metro regions (81.8% vs. 90.0% and 95.7%). Median survival time was 15.2 months for NH-Black, 19.1 months for NH-White, and 17.6 months for Hispanic patients. For those without a documented death in the NCDB, median time to last encounter was 38.3 months for NH-Black, 52.0 months for NH-White, and 42.7 months for Hispanic patients.

**Figure 1:**
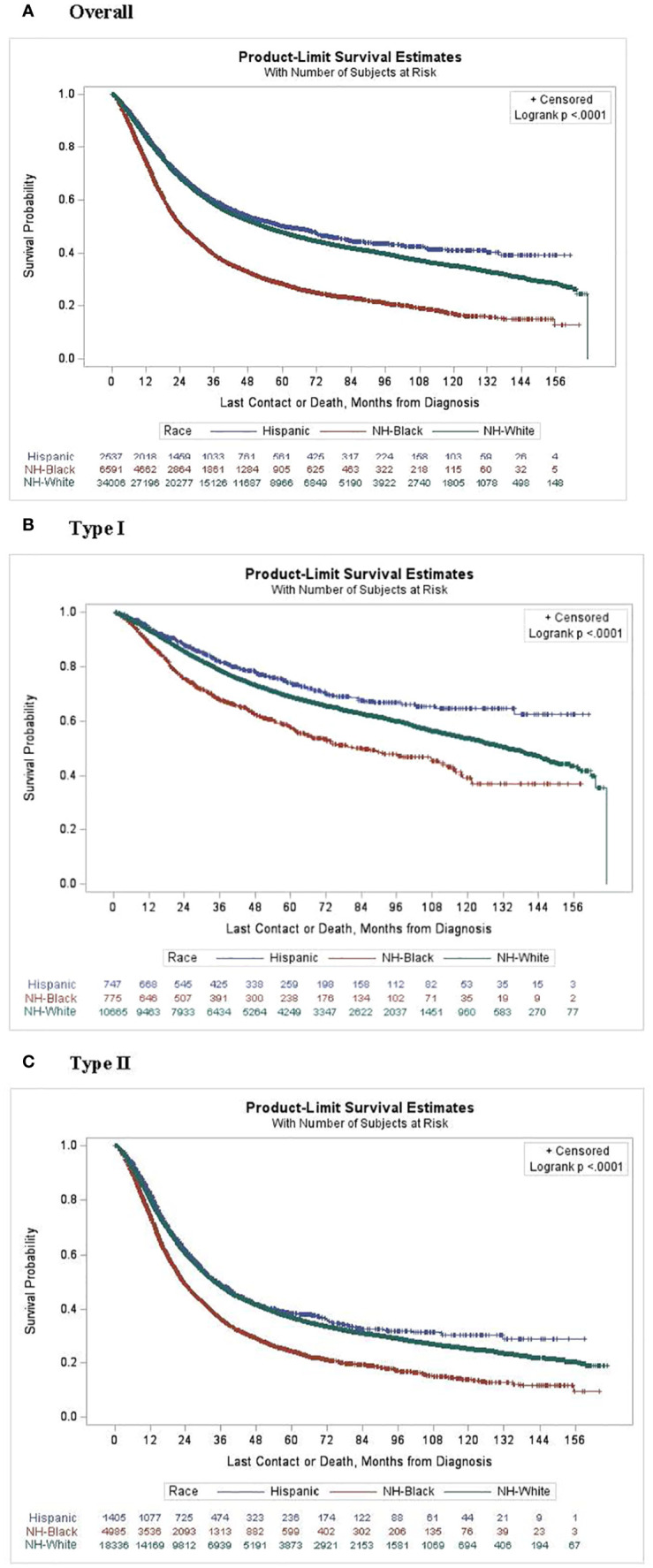
Kaplan-Meier curves for survival probability by race and subtype. **(A)** Overall, **(B)** Type 1 and **(C)** Type 2.

Kaplan-Meier estimates by race (**Figure 1**) indicated that NH-White and Hispanic patients had better survival compared with NH-Black patients (log-rank P<0.0001). In unadjusted Cox proportional hazards models on all-cause mortality risk (**Table 2**), NH-Black patients had a significantly higher risk of death than NH-White patients (HR: 1.71; 95% CI: 1.65, 1.77). This association was attenuated but persisted after adjusting for clinical and demographic covariates (HR. 1.30; 95% CI: 1.26, 1.35). NH-Black patients had a significantly worse adjusted survival than NH-White patients among those with both type I (HR 1.42; 95% CI: 1.26, 1.59) and type II (HR 1.28; 95% CI: 1.24, 1.34) disease. In analyses stratified by facility type, racial disparities persisted in academic (Black-White HR: 1.41, 95% CI: 1.34, 1.48) and non-academic (Black-White HR: 1.54, 95% CI: 1.47, 1.62) settings, although the disparity was larger in non-academic facilities (**Table 3**).

**Table 2:** Cox proportional hazards models for racial disparities in survival among advanced stage uterine cancer patients who received chemotherapy and/or radiotherapy, stratified by subtype.

	n/N	person-months	Model 1^a^	Model 2^b^
Overall				
NH-White	17155/34006	1465874.2	REF	REF
NH-Black	4341/6591	199298.3	**1.71 (1.65, 1.77)**	**1.30 (1.26, 1.35)**
Hispanic	1117/2537	100780.4	**0.92 (0.87, 0.98)**	**0.92 (0.87, 0.98)**
Type I				
NH-White	3443/10665	599874.0	REF	REF
NH-Black	320/775	36078.5	**1.52 (1.35, 1.70)**	**1.42 (1.26, 1.59)**
Hispanic	177/747	39156.5	**0.78 (0.67, 0.91)**	0.88 (0.76, 1.03)
Type II				
NH-White	11117/18336	696996.2	REF	REF
NH-Black	3450/4985	144504.3	**1.40 (1.35, 1.46)**	**1.28 (1.24, 1.34)**
Hispanic	754/1405	48565.5	0.94 (0.88, 1.01)	**0.93 (0.86, 1.00)**

a Unadjusted.

b Adjusted for age, year of diagnosis, Charlson-Deyo comorbidity score, cancer stage, surgery receipt, chemotherapy receipt, radiotherapy receipt, and subtype (not included in stratified models).

n indicates number of deaths; N, total number of individuals.

Bold = statistically significant with p<0.05 (confidence interval does not cross 1)

**Table 3:** Cox proportional hazards models for racial disparities in survival among advanced stage uterine cancer patients who received chemotherapy and/or radiotherapy, stratified by facility type.

	n/N	person-months	Model 1^a^	Model 2^b^
Academic				
NH-White	7038/13965	594975.4	REF	REF
NH-Black	2352/3624	110834.9	**1.66 (1.58, 1.73)**	**1.41 (1.34, 1.48)**
Hispanic	597/1353	55546.6	**0.89 (0.82, 0.97)**	**0.90 (0.83, 0.98)**
Not Academic				
NH-White	10117/20041	870898.8	REF	REF
NH-Black	1989/2967	88563.4	**1.76 (1.68, 1.85)**	**1.54 (1.47, 1.62)**
Hispanic	520/1184	45233.8	0.96 (0.88, 1.04)	1.01 (0.93, 1.10)

a Unadjusted.

b Adjusted for age, year of diagnosis, Charlson-Deyo comorbidity score, cancer stage, surgery receipt, chemotherapy receipt, radiotherapy receipt, and subtype (not included in stratified models).

n indicates number of deaths; N, total number of individuals.

Bold = statistically significant with p<0.05 (confidence interval does not cross 1)

In our analysis of HCA (**Table 4**), NH-Black patients had significantly worse survival compared to NH-White patients after adjusting for demographic/clinical factors and each HCA dimension individually. Living in high-income areas, a measure of higher affordability, was associated with 6% lower risk of death (HR: 0.94; 95% CI: 0.90, 0.99), and having private insurance versus no insurance was associated with 19% lower risk of death (HR: 0.81; 95% CI: 0.75, 0.86). Receiving treatment at an academic facility, a measure of higher availability, was associated with 6% reduced risk of death (HR: 0.94; 95% CI: 0.92, 0.97). Area of residence and crowfly distance, measures of accessibility, were not associated with differences in survival. In models adjusted for all three HCA domains, NH-Black patients similarly had a significantly higher risk of death than NH-White patients (HR 1.29; 95% CI: 1.24, 1.34), and Hispanic patients had a lower risk (HR 0.91; 95% CI: 0.85, 0.97). The proportional hazards assumption was met for race/ethnicity.

**Table 4:** Cox proportional hazards models for racial disparities in survival among advanced stage uterine cancer patients who received chemotherapy and/or radiotherapy, adjusted for healthcare access variables.

	Clinical^a^ + Affordability^b^	Clinical^a^ + Availability^c^	Clinical^a^ + Accessibility^d^	Clinical^a^ + All Three^b,c,d^
Race (ref: NH-White)				
NH-Black	**1.28 (1.23, 1.33)**	**1.32 (1.27, 1.36)**	**1.31 (1.27, 1.35)**	**1.29 (1.24, 1.34)**
Hispanic	**0.90 (0.85, 0.96)**	**0.93 (0.88, 0.99)**	**0.93 (0.87, 0.99)**	**0.91 (0.85, 0.97)**
Income (ref: <$40,227)				
$40,227-$50,353	1.01 (0.97, 1.06)			1.01 (0.97, 1.05)
$50,354-$63,332	1.01 (0.97, 1.06)			1.01 (0.97, 1.06)
$63,333+	**0.94 (0.90, 0.99)**			**0.95 (0.90, 1.00)**
Education (ref: >17.6%)				
10.9-17.5%	1.02 (0.98, 1.06)			1.02 (0.98, 1.06)
6.3-10.8%	1.01 (0.96, 1.05)			1.00 (0.96, 1.05)
<6.3%	1.00 (0.95, 1.05)			0.99 (0.94, 1.05)
Insurance (ref: None)				
Government	**0.89 (0.83, 0.95)**			**0.88 (0.82, 0.95)**
Private	**0.81 (0.75, 0.86)**			**0.80 (0.75, 0.86)**
Facility Type (ref: Not Academic)				
Academic		**0.94 (0.92, 0.97)**		**0.94 (0.92, 0.97)**
Crowfly			1.00 (1.00, 1.00)	1.00 (1.00, 1.00)
Residence (ref: Metro)				
Urban			1.03 (1.00, 1.07)	0.99 (0.96, 1.03)
Rural			1.06 (0.96, 1.17)	1.01 (0.92, 1.12)

a Adjusted for age, Charlson-Deyo comorbidity score, subtype, year of diagnosis, surgery receipt, chemotherapy receipt, radiotherapy receipt, and cancer stage.

b Adjusted for insurance, education, and income.

c Adjusted for facility type.

d Adjusted for crowfly and residence.

Bold = statistically significant with p<0.05 (confidence interval does not cross 1)

## Discussion

### Summary of main results

In this retrospective study of patients with stage III-IV uterine cancer, NH-Black patients were more likely to have stage IV disease and type II cancers and had a significantly higher risk of death compared to NH-White patients regardless of histologic subtype. NH-Black patients were more likely to have public insurance and live in neighborhoods with lower median income than NH-White patients. Higher affordability measures were associated with lower risk of death. Importantly, NH-Black patients had significantly increased risk of death compared NH-White patients even after adjusting for demographic, clinical, and HCA variables.

### Results in the context of published literature

Our findings describing racial disparities in uterine cancer align with results from prior studies (8, 9, 22). Black women were more likely to have aggressive histologic variants and worse survival compared to White women after stratifying by age, grade, stage, and histologic subtype (9, 17, 22). Recent advances in uterine cancer research suggest that molecular classification may improve our understanding of prognosis compared to the historical pathogenic subtypes (23). However, large databases do not capture genomic data; thus, we divided patients by histologic subtypes to approximate low- versus high-risk disease. NH-Black patients were more likely to receive chemotherapy only and less likely to receive chemoradiation than NH-White patients despite having more aggressive cancers. This disparity is significant given our current knowledge of recommended treatment for high-risk uterine cancer. A recent study on patients with high-risk uterine cancer found that molecular classification had strong prognostic value, and patients with p53 abnormal tumors had significantly longer disease-free survival with adjuvant chemoradiation compared to radiotherapy alone (24). Because NH-Black patients are more likely to have high risk histologic subtypes and mutations, including molecular classification in large databases may allow for better evaluation and treatment.

We evaluated how multiple dimensions of healthcare access contribute to racial and ethnic disparities in uterine cancer survival. Measures of affordability, including insurance status and income, were associated with survival. Patients who lived in the highest income areas had lower risk of death, similar to prior findings (25). Patients with private insurance had lower risk of death compared to those with no insurance. In prior studies, having public or no insurance was associated with advanced stage disease at diagnosis, insufficient treatment, and worse survival (26). These results are consistent with our findings among patients with ovarian cancer; higher affordability was associated with receipt of guideline-concordant treatment (27). Our results highlight the importance of improving access to affordable, high-quality preventive, diagnostic, and treatment services for patients with uterine cancer.

We found that patients who received treatment at an academic center had lower risk of death, and NH-Black patients were more likely to receive care at academic centers. Despite this, NH-Black patients had worse survival compared to NH-White patients in both unadjusted and adjusted models. A prior study similarly found that Black women were more likely to be treated at academic centers, but twice as likely to die compared to White women, and proposed that cancer stage and grade contributed to nearly all of the racial difference in survival (28). We found no differences in survival by HCA accessibility, represented by area of residence and crowfly distance. NH-Black patients were more likely to live in metropolitan areas and had shorter distances to hospital sites than NH-White patients. These findings demonstrate that improved HCA accessibility may not correlate with greater healthcare utilization. For example, NH-Black and Hispanic patients who live in metro or urban areas may have additional challenges to accessing healthcare including lack of transportation and lack of access to internet or telephone services for appointment scheduling. While rural residence was not associated with worse survival in our study, less than 2% of our cohort resided in a rural area, and White patients were more likely to reside in rural areas. Studies have shown that patients with lung and breast cancer who reside in a rural areas were less likely to receive guideline-concordant care, more likely to experience treatment delays, and had worse survival compared to patients living in urban areas (29, 30). There are no studies investigating outcomes in uterine cancer among rural areas, and additional research on this group is needed.

Hispanic patients had the lowest risk of death after adjusting for demographic, clinical, and HCA variables despite having HCA profiles similar to NH-Black patients and being the least likely to have insurance and most likely to have low area-level education. This “Hispanic paradox” complicates the role of SES on disparities in mortality (31). However, studies have shown that the association between Hispanic ethnicity and improved survival loses significance after adjusting for tumor characteristics and treatment (32). Other studies suggest that this paradox can be explained by the finding that foreign-born Hispanic females without cancer have reduced mortality rates (33). However, the NCDB does not distinguish between US-born and foreign-born Hispanic women to investigate this theory in our study population. The mechanisms driving this paradox are not well-defined and require further exploration.

### Strengths and limitations

There are several limitations of this study. The NCDB is a hospital-based dataset and is not designed to represent the entire US population, limiting the generalizability of our results. Many affordability measures utilized were calculated at the area rather than individual level and may not accurately reflect one’s unique circumstances. We were limited by the HCA variables available in the NCDB. We used histologic subtype as a measure of low versus high risk; molecular classification may offer unique insights in determining prognosis and treatment but is not available in the NCDB. Lastly, the NCDB does not account for clinical trials or targeted therapy, which could significantly impact survival. Despite these limitations, the NCDB allows for use of a large sample size over a significant period to identify associations between HCA and survival.

### Implications for practice and future research

NH-Black patients had significantly greater risk of death compared to NH-White patients after accounting for clinical, demographic, and HCA variables. Thus, we must address factors beyond these dimensions to bridge survival disparities. This study did not account for two dimensions of healthcare access: accommodation and acceptability. While difficult to measure in large databases, these dimensions encompass measures of intercultural competence and unconscious bias, which are important mediators between HCA and survival. A patient’s perception or experience of discrimination is associated with decreased use of healthcare services and decreased adherence to recommendations from medical providers (34, 35). In a qualitative study on Black women with uterine cancer, subjects described knowledge deficits, misinterpretations of symptoms, and misaligned responses from providers as contributors to delays in care (36). Other gynecologic disparities by race may also contribute to our findings. Compared to White women, Black women are twice as likely to have a hysterectomy prior to menopause, leading to an underestimate of the true incidence of uterine cancer among Black women (37). Future studies on accommodation and acceptability in uterine cancer are needed to fully describe the association between HCA and survival.

In conclusion, health care access affordability and availability were associated with improved survival for patients with advanced stage uterine cancer. Compared to NH-White patients, NH-Black patients had worse survival regardless of pathogenic subtype after adjusting for demographic, clinical, and HCA variables. Future research on additional HCA dimensions and barriers to quality care are necessary to improve the racial gap in survival for patients with uterine cancer.

## Data availability statement

The original contributions presented in the study are included in the article/supplementary material. Further inquiries can be directed to the corresponding author.

## Author contributions

MA: Conceptualization, Data curation, Formal Analysis, Funding acquisition, Investigation, Methodology, Project administration, Resources, Software, Supervision, Validation, Visualization, Writing – original draft, Writing – review & editing. AG: Writing – original draft, Writing – review & editing, Conceptualization, Data curation, Formal Analysis, Investigation, Methodology, Supervision. TA: Conceptualization, Investigation, Methodology, Project administration, Resources, Supervision, Writing – review & editing. RP: Conceptualization, Investigation, Methodology, Project administration, Resources, Supervision, Visualization, Writing – review & editing.
